# Effects of Arbuscular Mycorrhizal Fungi on the Growth and Nutrient Uptake in Wheat Under Low Potassium Stress

**DOI:** 10.3390/plants14091288

**Published:** 2025-04-24

**Authors:** An-Qi Han, Shuai-Bo Chen, Dan-Dan Zhang, Jin Liu, Meng-Chuan Zhang, Bin Wang, Yue Xiao, Hai-Tao Liu, Tian-Cai Guo, Guo-Zhang Kang, Ge-Zi Li

**Affiliations:** 1 The National Engineering Research Center for Wheat, Henan Agricultural University, Zhengzhou 450046, China; ymy2549079816@163.com (A.-Q.H.); ccc3192@stu.henau.edu.cn (S.-B.C.); zdd13592603204@163.com (D.-D.Z.); mengchuanzhang@163.com (M.-C.Z.); wangbzmx@163.com (B.W.); 15831079657@163.com (Y.X.); 2 Henan Technological Innovation Centre of Wheat, Henan Agricultural University, Zhengzhou 450046, China; liujin@stu.henau.edu.cn; 3 College of Resources and Environment, Henan Agricultural University, Zhengzhou 450002, China; liuhaitaoky@henau.edu.cn; 4 State Key Laboratory of High-Efficiency Production of Wheat-Maize Double Cropping, Henan Agricultural University, Zhengzhou 450046, China

**Keywords:** wheat, arbuscular mycorrhizal fungi, nutrient uptake, low potassium stress

## Abstract

Potassium (K) plays important roles in plant growth and development processes, while low K (LK) stress inhibits plant growth by altering reactive oxygen species accumulation. Arbuscular mycorrhizal fungi (AMF) promote nutrient absorption and transport in plants. However, the roles of AMF in affecting K nutrition are less well studied than those of other nutrients, especially in wheat. In this study, the effects of AMF on four wheat varieties were evaluated; results showed that the inoculation with the AMF-*Rhizophagus intraradices* significantly increased mycorrhizal colonization, fresh and dry weights, ascorbic acid, and glutathione contents, while decreasing malondialdehyde contents under both normal and LK stress treatments. It is worth noting that the contents of K and several nutrient elements were more significantly increased in roots than in shoots, suggesting that AMF mainly affect the uptake of K and other nutrient elements in the roots. Moreover, the expression levels of *K transporter* genes were higher than those of *nitrogen* and *phosphorus transporter* genes, especially under AMF combined with LK stress treatments. These results indicate that AMF improves wheat growth and antioxidant activity by regulating K transporter gene expression and affecting K uptake and transport. Therefore, AMF could be used as a sustainable agricultural alternative in wheat under LK soils.

## 1. Introduction

Potassium (K) is one of the three major macronutrients. It plays an essential role in the growth, development, and different metabolic processes of plants [1,2,3], including wheat [4,5,6]. It has been reported that K deficiency has been occurring in soil in different agricultural areas in the world [7]. With the progress of climate change, K availability has become reduced: in China, K utilization rates in wheat are generally lower by 45% [8]. Thus, there is an urgent need to develop effective strategies to improve K utilization rates for the purposes of sustainable agriculture [9].

Arbuscular mycorrhizal fungi (AMF) are beneficial soil microorganisms that mostly combine with plant roots to form symbiotic associations [10,11]. Previous studies have reported that AMF play a crucial role in increasing plant growth under different agricultural environmental stresses by increasing the uptake of mineral nutrients, such as nitrogen (N), phosphorus (P), K, and sulfur (S) [12,13,14,15]. It has been reported that AMF, as bio-fertilizers, can be used to reduce or replace inorganic chemical fertilizers in agricultural practices [9,16,17].

Wheat (*Triticum aestivum* L.) is one of the most important food crops in the world [18]. Thus, the yield and quality of wheat are important for the needs of humans. However, the high K inputs and low K use efficiency in the agricultural system have restricted the wheat yields and further quality improvements [4,7]. Moreover, low K (LK) and K deficiency (DK) stress decreases growth and dry mass accumulation in wheat [5,19].

AMF have been reported to increase plant growth, biomass, and grain yields under different forms of stress, such as drought stress and salt stress, by improving the uptake of various nutrients and ion homeostasis [16,20,21]. A large number of studies have reported that AMF can improve the absorption and transport of N and P in different plants, such as in wheat [22,23]. Zhu et al. [24] found that AMF increases N uptake and N use efficiency in wheat grown at elevated CO_2_ levels; Ingraffia et al. [25] found that inoculation of AMF promotes N and P absorption under P-limited conditions; Xue et al. [26] found that inoculation of AMF increases root N uptake from fertilizers and the soil. To our knowledge, however, few studies have reported that AMF improves K nutrition uptake in plants [27,28], especially in wheat, and there are still no reports on the effects of AMF inoculation on K uptake under K stress.

The aim of this study was to investigate the effect of inoculation with the AMF of *Rhizophagus intraradices* (RI), which was reported in a previous article [29], on the uptake of K and other mineral elements, transport-related gene expression, and antioxidants in four wheat varieties under LK stress treatments. This study hypothesizes that AMF inoculation promotes wheat growth by increasing the antioxidant response, improving K uptake and that of other mineral elements, and changing the uptake and expression of transport-related genes under LK stress treatments.

## 2. Results

### 2.1. AMF Colonization Under Low Potassium Stress

**Figure 1:**
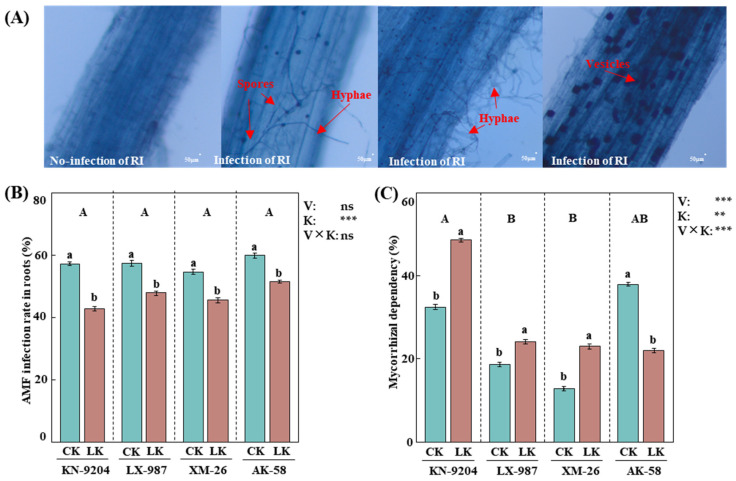
Effects of AMF colonization in four wheat varieties. (**A**) Acid-ink staining of wheat root after AMF colonization, bar = 50 μm. (**B**) AMF colonization rate in the root of four wheat varieties under normal potassium (CK) and low potassium (LK) stress treatments. (**C**) Mycorrhizal dependency of AMF in the root of four wheat varieties under CK and LK stress treatments. AMF of *Rhizophagus intraradices* (RI) were applied in this study. Four wheat varieties were Kenong 9204 (KN-9204), Lunxuan 987 (LX-987), Xinmai 26 (XM-26), and Aikang 58 (AK-58). Asterisks indicate significant differences in variety (V) and K level (K) and their interactions [Kruskal–Wallis test, *n* = 3, *p* < 0.01 (**), *p* < 0.001 (***) and *p* > 0.05 (ns)]. Data present means ± SE (*n* = 3). Different letters are used to indicate significant differences; the small letters indicate differences between treatments within each variety, and the capital letters indicate differences between varieties (Tukey’s test, *n* = 3, *p* < 0.05).

To explore the roles of AMF in wheat, the AMF-RI (*Rhizophagus intraradices*) were inoculated in soil under normal (CK) and LK stress treatments. After 30 days of RI inoculation, the AMF structures in the roots were scanned using a stereomicroscope. The results showed the presence of vesicles, hyphae, and spores in the roots of wheat inoculated with RI, which were not present in the controls with no RI inoculation, suggesting that RI establishes a symbiotic relationship with wheat (Figure 1A). Moreover, AMF colonization was measured in the roots of four wheat varieties: KN-9204 (Kenong 9204), LX-987 (Lunxuan 987), XM-26 (Xinmai 26), and AK-58 (Aikang 58). It was found that the respective values of AMF colonization in the above four wheat varieties under the CK and LK treatments were 57.4% and 42.8%, 57.5% and 48.0%, 54.7% and 45.6%, and 59.9% and 51.6% (Figure 1B). Furthermore, the values of mycorrhizal dependence were 32.5% and 48.6% in KN-9204, 18.7% and 24.1% in LX-987, 12.9% and 23.0% in XM-26, and 38.7% and 22.0% in AK-58 under the CK and LK treatments (Figure 1C), respectively. Interaction analysis found that the colonization rate of AMF was not significantly different among the four wheat varieties (Figure 1B), while the mycorrhizal dependency did differ significantly, particularly in KN-9204 (Figure 1C).

### 2.2. AMF Improve Wheat Growth

**Figure 2:**
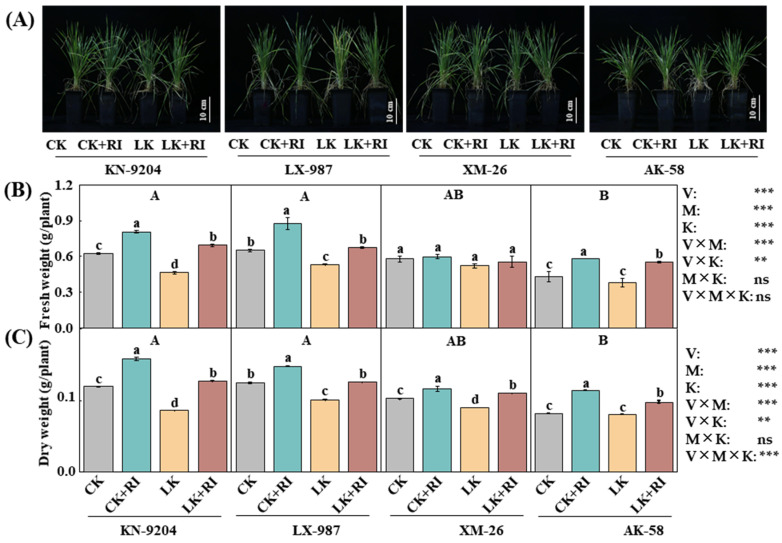
Effects of AMF-RI colonization on the growth of four wheat varieties under CK and LK stress. (**A**) The phenotype of four wheat varieties after AMF-RI colonization for 30 d, bar = 10 cm. (**B**) Fresh weights of different treatments after AMF-RI colonization for 30 d. (**C**) Dry weights of different treatments after AMF-RI colonization for 30 d. CK, normal potassium; CK + RI, CK added with *Rhizophagus intraradices* (RI); LK, low potassium; LK + RI, LK added with RI. Four wheat varieties were Kenong 9204 (KN-9204), Lunxuan 987 (LX-987), Xinmai 26 (XM-26), and Aikang 58 (AK-58). Asterisks indicate significant differences in variety (V), AMF inoculation (M), K level (K), and their interactions [Kruskal–Wallis test, *n* = 3, *p* < 0.01 (**), *p* < 0.001 (***) and *p* > 0.05 (ns)]. Data present means ± SE (*n* = 3). Different letters are used to indicate significant differences; the small letters indicate differences between treatments within each variety, and the capital letters indicate differences between varieties (Tukey’s test, *n* = 3, *p* < 0.05).

To explore the effects of AMF on wheat growth, the phenotypes of the four wheat varieties were recorded on days 10–30. The results indicated that, after RI inoculation, the growth phenotypes of all four wheat varieties under CK and LK treatments were significantly improved (Figure 2A). Compared with the CK treatment alone, the dry and fresh weights increased by 29.4% and 32.5% in KN-9204, 34.4% and 18.7% in LX-987, 3.4% and 12.8% in XM-26, and 35.2% and 38.7% in AK-58 under the CK + RI treatments (CK with added RI) (Figure 2B,C). Under the LK + RI treatments (LK with added RI), increases of 50.0% and 48.6%, 27.1% and 24.1%, 7.1% and 23.0%, and 45.5% and 22.0% were recorded, relative to LK alone (Figure 2B,C). Moreover, interaction analysis found that the fresh and dry weights were significantly different by wheat variety (V), AMF inoculation (M), and K level (K). These results suggest that RI inoculation significantly improved wheat growth under both CK and LK treatments, particularly in KN-9204.

### 2.3. AMF Affect the Antioxidant Defense of Wheat Seedlings

**Figure 3:**
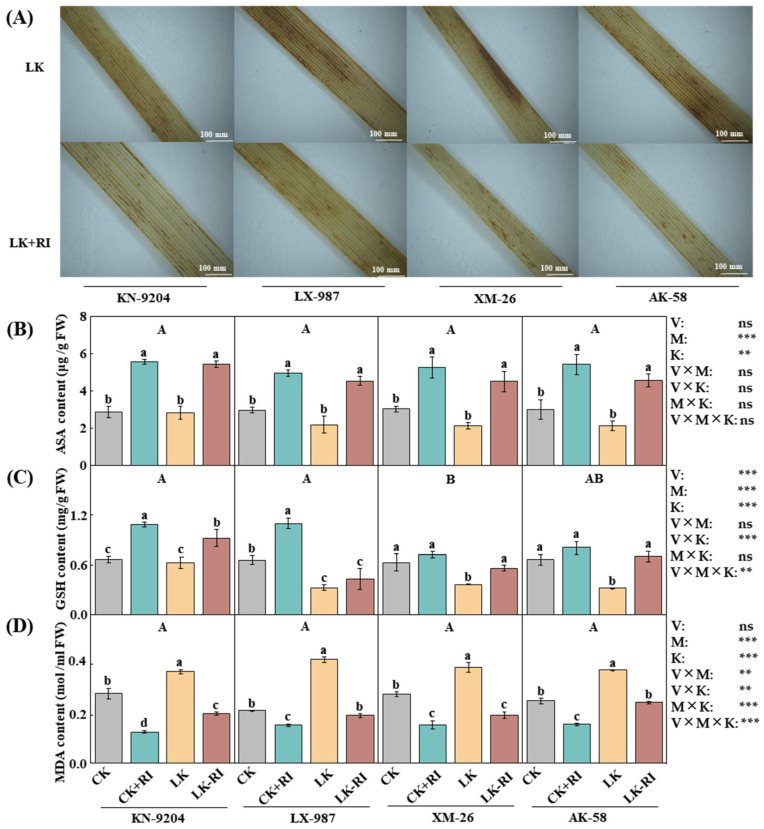
Effects of AMF-RI colonization on antioxidant capacity of four wheat varieties under CK and LK stress treatments. (**A**) DAB staining of the peroxide (H_2_O_2_) at 30 d after AMF-RI colonization, bar = 100 mm. (**B**) ASA content at 30 d after AMF-RI colonization. (**C**) GSH content at 30 d after AMF-RI colonization. (**D**) MDA content at 30 d after AMF-RI colonization. CK, normal potassium; CK + RI, CK added with *Rhizophagus intraradices* (RI); LK, low potassium; LK + RI, LK added with RI. Four wheat varieties were Kenong 9204 (KN-9204), Lunxuan 987 (LX-987), Xinmai 26 (XM-26), and Aikang 58 (AK-58). Asterisks indicate significant differences in variety (V), AMF inoculation (M), K level (K), and their interactions [Kruskal–Wallis test, *n* = 3, *p* < 0.01 (**), *p* < 0.001 (***) and *p* > 0.05 (ns)]. Data present means ± SE (*n* = 3). Different letters are used to indicate significant differences; the small letters indicate differences between treatments within each variety, and the capital letters indicate differences between varieties (Tukey’s test, *n* = 3, *p* < 0.05).

To identify the roles of RI in wheat antioxidant defense, antioxidant substances, such as ascorbate (ASA), glutathione (GSH), malondialdehyde (MDA), and hydrogen peroxide (H_2_O_2_), were measured in wheat under different treatments. DAB (3,3-diaminobenzidine) staining showed that the leaf color, which represents H_2_O_2_ content, was significantly changed in the leaves of the four wheat varieties and was darker in no-RI-inoculated group than in the RI inoculation treatment (Figure 3A). Moreover, under the CK + RI condition, ASA content was significantly increased by 93.8%, 66.9%, 74.6%, and 80.6% in KN-9204, LX-987, XM-26, and AK-58; it was even more significantly increased under the LK + RI condition, with increments of 94.2%, 107.5%, 114.7%, and 116.1% (Figure 3B), respectively. Interaction analysis found that the ASA contents differed significantly by AMF inoculation and K level but not by wheat variety (Figure 3B). Similarly, under the CK + RI condition, GSH content increased significantly—by 62.5% and 67.2%—in KN-9204 and LX-987, while there were no changes in XM-26 and AK-58 (Figure 3C). Conversely, under the LK + RI condition, GSH contents were significantly increased in these latter two varieties—by 54.4% and 122.8%, respectively—but they were not changed in KN-9204 and LX-987 (Figure 3C). Interaction analysis found that GSH contents differed significantly by wheat variety, AMF inoculation, and K level (Figure 3C). Furthermore, under the CK + RI condition, MDA content was significantly decreased by 58.5%, 27.5%, 44.5%, and 37.7% in KN-9204, LX-987, XM-26, and AK-58; it was also significantly decreased under the LK + RI treatments, with decreases of 45.8%, 54.0%, 49.7%, and 34.3% (Figure 3D), respectively. Interaction analysis found that MDA content differed significantly by AMF inoculation and K level but not by wheat variety (Figure 3D).

### 2.4. AMF Enhance the Uptake and Transport of K and Several Mineral Elements

**Figure 4:**
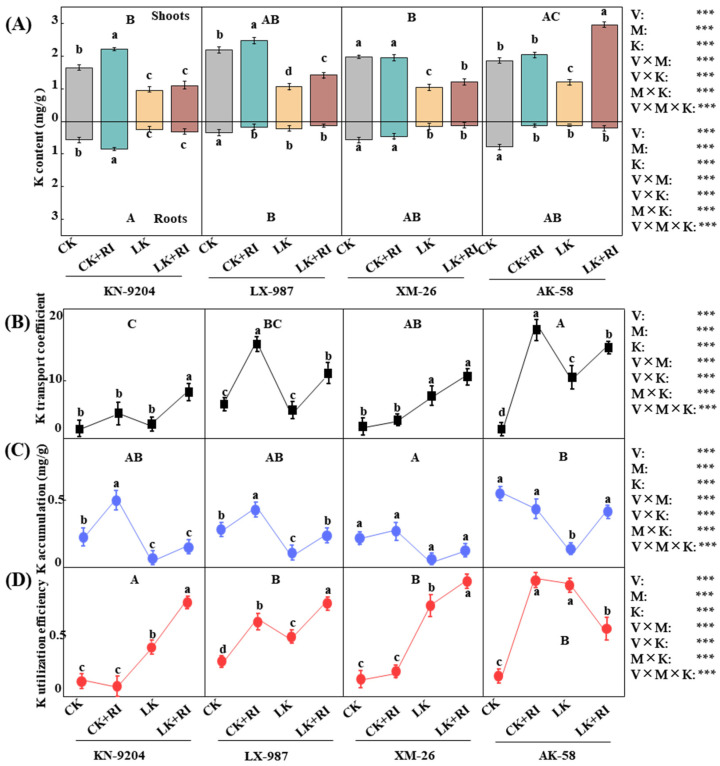
Effects of AMF-RI colonization on the K uptake of four wheat varieties under CK and LK stress treatments. (**A**) K content in four wheat varieties at 30 d after AMF-RI colonization. (**B**) K transport coefficient in four wheat varieties at 30 d after AMF-RI colonization. (**C**) K accumulation in four wheat varieties at 30 d after AMF-RI colonization. (**D**) K utilization efficiency in four wheat varieties at 30 d after AMF-RI colonization. CK, normal potassium; CK + RI, CK added with *Rhizophagus intraradices* (RI); LK, low potassium; LK + RI, LK added with RI. Four wheat varieties were Kenong 9204 (KN-9204), Lunxuan 987 (LX-987), Xinmai 26 (XM-26), and Aikang 58 (AK-58). Asterisks indicate significant differences in variety (V), AMF inoculation (M), K level (K), and their interactions [Kruskal–Wallis test, *n* = 3, *p* < 0.001 (***)]. Data present means ± SE (*n* = 3). Different letters are used to indicate significant differences; the small letters indicate differences between treatments within each variety, and the capital letters indicate differences between varieties (Tukey’s test, *n* = 3, *p* < 0.05).

To explain the roles of RI in the uptake and transport of K and other mineral elements, their contents were measured in the studied wheat plants. The results showed that, under the CK + RI condition relative to CK, K content was increased by 35.0%, 13.4%, and 9.3% in the leaves of KN-9204, LX-987, and AK-58, but it did not change in XM-26, and in the roots, it was only increased in KN-9204, by 48.4% (Figure 4A). Compared with LK, under LK + RI treatments, K content increased by 14.7%, 31.6%, 14.1%, and 144.7% in the leaves of KN-9204, LX-987, XM-26, and AK-58; in the roots, it decreased by 53.0% and 20.7% in LX-987 and XM-26, while in KN-9204 and AK-58, it was increased by 43.5% and 67.6% (Figure 4A). Moreover, the K transport coefficient and K accumulation were increased by 115.1%, 106.6%, 41.3%, and 46.1% and 131.7%, 75.6%, 53.5%, and 151.9% in these four wheat varieties under the LK + RI condition compared with CK and LK treatment only. Under the CK + RI condition, the K transport coefficient was increased by 141.3%, 24.8%, and 66.7% in LX-987, XM-26, and AK-58, and K accumulation was increased by 96.4%, 42.9%, and 17.2% in KN-9204, LX-987, and XM-26 (Figure 4B), respectively. K utilization efficiency increased by 112.8%, 26.1%, and 60.1% in LX-987, XM-26, and AK-58 and decreased by 32.6% in KN-9204 under the CK + RI condition, while it increased by 87.5%, 57.0%, and 23.8% in KN-9204, LX-987, and XM-26, respectively, under the LK + RI condition (Figure 4D). Furthermore, interaction analysis found that K content, the K transport coefficient, K accumulation, and K utilization efficiency were significantly different by wheat variety, AMF inoculation, and K level (Figure 4).

In addition to K, the contents of other elements, such as P (phosphorus), Ca (calcium), and Mg (magnesium), were measured in this study. The results indicated that the P content in the roots and shoots was increased in both LX-987 (51.1% and 13.1%) and AK-58 (23.6% and 44.3%) under the CK + RI condition compared with CK only, as well as in KN-9204 (29.1% and 15.2%), LX-987 (58.7% and 9.5%), and XM-26 (22.3% and 8.8%) under the LK + RI condition compared with LK only (Table A2). Moreover, the P transport coefficient was increased by 59.1%, 33.7%, and 25.1% in KN-9204, LX-987, and XM-26, as well as by 12.1%, 45.0%, 12.4%, and 62.8% in KN-9204, LX-987, XM-26, and AK-58 under the CK + RI and LK + RI treatments (Table A2), respectively. Interaction analysis found that P content in the shoots was significantly different by wheat variety, AMF inoculation, and K level, but the P transport coefficient was significantly different by K level only (Table A3).

Similarly, differences in the contents of Ca and Mg were observed in the roots and shoots of the four wheat varieties (Table A2). Under the CK + RI condition, Ca content increased in XM-26 (9.3% in the shoots and 17.5% in the roots) and AK-58 (19.9% in the shoots and 178.7% in the roots), and the Ca transport coefficient decreased by 21.5%, 67.0%, 6.9%, and 57.0% in the four wheat varieties (Table A2). Mg content increased in XM-26 (15.2% and 11.4% in the shoots and roots) and AK-58 (14.9% and 42.6%), but the Mg transport coefficient decreased by 17.1%, 32.1%, and 19.4% in KN-9204, LX-987, and AK-58 (Table A2), respectively. Under the LK + RI condition, Ca content increased by 12.1% and 10.5% in the shoots of KN-9204 and LX-987 and by 52.0%, 14.3%, 80.8%, and 185.5% in the roots of KN-9204, LX-987, XM-26, and AK-58, while the Ca transport coefficient decreased by 26.2%, 3.4%, 43.7%, and 77.5% (Table A2), respectively. Mg content increased by 10.1%, 6.2%, and 10.8% in the shoots of KN-9204, LX-987, and XM-26, as well as by 35.1%, 48.9%, and 45.0% in the roots of LX-987, XM-26, and AK-58; the Mg transport coefficient decreased by 21.4%, 25.6%, and 34.4%, respectively, in LX-987, XM-26, and AK-58 (Table A2). Interaction analysis found that Ca and Mg contents in the roots, as well as the Ca and Mg translocation coefficients, were significantly different (*p* < 0.001) by AMF inoculation and K level, while Ca content in the roots and the Mg translocation coefficient were also significantly different (*p* < 0.01) by wheat variety (Table A3). All these results suggest that AMF affect the uptake and transport of mineral elements in these four wheat varieties, especially those of K and P in KN-9204.

### 2.5. Correlation Analysis Between AMF and Various Indices in Four Wheat Seedlings

**Figure 5:**
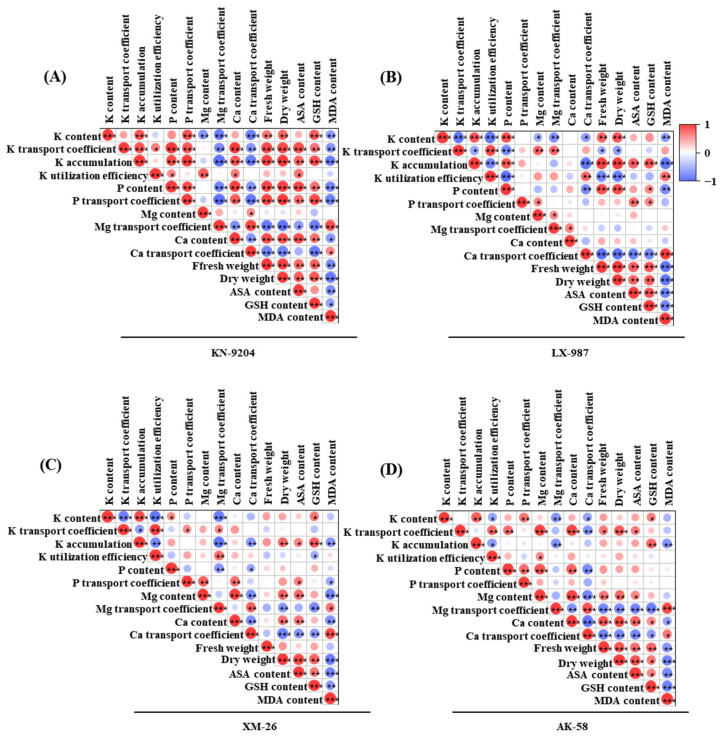
Correlation analysis of different physiological indicators in four wheat varieties after AMF-RI colonization under CK and LK stress treatments. (**A**) Correlation analysis of different indicators in KN-9204. (**B**) Correlation analysis of different indicators in LX-987. (**C**) Correlation analysis of different indicators in XM-26. (**D**) Correlation analysis of different indicators in AK-58. Gradient color bars denote the correlation coefficient between physiological indexes. The asterisks indicate a significant correlation in the data [*p* < 0.05 (*), *p* < 0.01 (**), *p* < 0.001 (***)].

The relationship between AMF and various indices in four wheat seedlings was determined using correlation analysis. The results showed that there were different correlations between the various induces in the four wheat plants (Figure 5). At the *p* ≤ 0.001 level, K content was significantly positively correlated with K accumulation, the P transport coefficient, and GSH content in KN-9204; K accumulation, P content, and dry weight in LX-987; and K accumulation in XM-26. It was also negatively correlated with the transfer coefficients of Mg and Ca in KN-9204; the K transport coefficient and K utilization efficiency in LX-987; and the K transport coefficient, K utilization efficiency, and Mg content in XM-26 (Figure 5A–D). In addition to the above indexes, at the *p* ≤ 0.01 level, K content was also significantly positively correlated with fresh and dry weight in KN-9204, fresh weight in LX-987, and K accumulation and the P transport coefficient in AK-58, and it was negatively correlated with MDA and Mg content in KN-9204, the Mg transport coefficient and MDA content in LX-987, and the Mg transport coefficient in AK-58 (Figure 5A–D). Moreover, at the *p* ≤ 0.05 level, K content was positively correlated with P content and GSH content in XM-26 and with GSH content in AK-58, and it was negatively correlated with Mg content and the Ca transport coefficient in LX-987 and with the Ca transport coefficient in AK-58 (Figure 5A–D). These results showed the following separate relationships with K content: nine indexes were significantly correlated for KN-9204, with five positively and four negatively correlated; ten indexes were significantly correlated for LX-987, four positively and six negatively; and finally, for XM-26 and AK-58, there were significant correlations with six indexes, three positively and three negatively (Figure 5A–D). Among these, K content was more significantly positively correlated with other indexes in KN-9204 than in the other three wheat varieties. Thus, KN-9204 was selected and used for further experiments.

### 2.6. AMF Affect the Expression Levels of Transporter Genes for K and Other Elements

**Figure 6:**
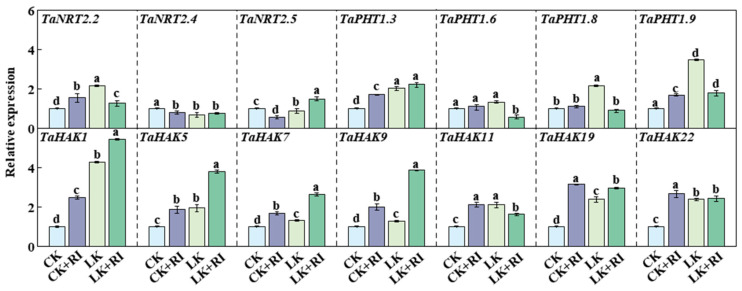
Effects of AMF-RI colonization on the expression levels of transporter genes in the roots of KN-9204 under CK and LK stress treatments. CK, normal potassium; CK + RI, CK added with *Rhizophagus intraradices* (RI); LK, low potassium; LK + RI, LK added with RI. Four wheat varieties were Kenong 9204 (KN-9204). Data present means ± SE (*n* = 3). Different letters are used to indicate significant differences (Tukey’s test, *n* = 3, *p* < 0.05).

To understand the molecular mechanism of how AMF regulate the uptake and transport of elements in wheat roots, several genes involved the transport and uptake of N (*TaNRT2.2*, *TaNRT2.4*, and *TaNRT2.5*), P (*TaPHT1.3*, *TaPHT1.6*, *TaPHT1.8*, and *TaPHT1.9*), and K (*TaHAK1*, *TaHAK5*, *TaHAK7*, *TaHAK9*, *TaHAK11*, *TaHAK19* and *TaHAK22*), as reported in previous studies [30,31,32], were selected and measured in this study. The results showed that the expression levels of several N and P transporter genes, such as *TaNRT2.2*, *TaPHT1.3*, and *TaPHT1.9*, and those of all K transporter genes (*TaHAK1*, *TaHAK5*, *TaHAK7*, *TaHAK9*, *TaHAK11*, *TaHAK19*, and *TaHAK22*), were significantly induced under the LK, CK + RI, and LK + RI treatments (Figure 6). Compared with CK, the K transporter genes *TaHAK1*, *TaHAK5*, *TaHAK7*, *TaHAK9*, *TaHAK11*, *TaHAK19*, and *TaHAK22* were significantly increased by 148.6%, 85.6%, 85.6%, 152.7%, 112.1%, 230.8%, and 166.0% under the CK + RI treatments and by 399.8%, 278.9%, 278.9%, 226.4%, 62.1%, 197.0%, and 141.8% under the LK + RI treatments (Figure 6), respectively. However, only the N transporter genes *TaNRT2.2* (increased by 54.2% and 27.0%) and the P transporter genes *TaPHT1.3* (increased by 70.4% and 122.1%) and *TaPHT1.9* (increased by 68.4% and 78.6%) were induced under both the CK + RI and LK + RI treatments (Figure 6). Among these genes, only the expression levels of *TaHAK1*, *TaHAK5*, *TaHAK7*, *TaHAK9*, and *TaPHT1.3* were more significantly increased under the LK + RI condition than under the CK + RI condition, suggesting that these genes play a crucial role in AMF’s improvement of the uptake and transport of elements under the LK stress treatments (Figure 6).

### 2.7. Analyses of AMF’s Effects on Changes in Various Indices in KN-9204

**Figure 7:**
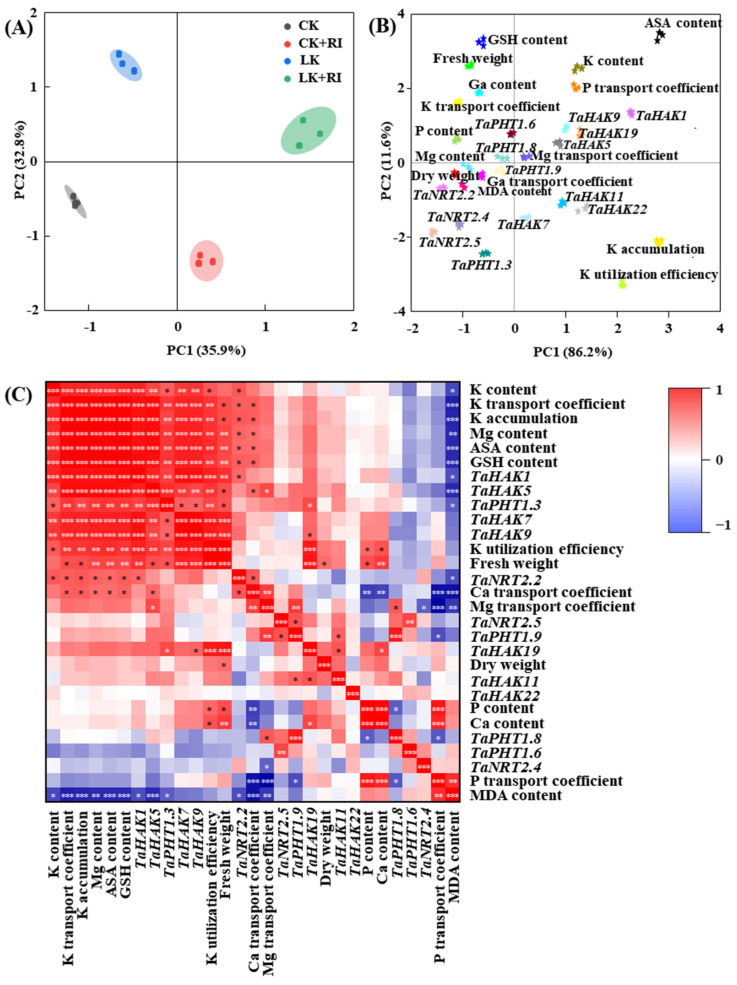
Principal component analysis (PCA) and Pearson correlation analyses of different indicators in KN-9204 after AMF-RI colonization under CK and LK stress treatments. (**A**) PCA of four treatments, which contained CK, CK + RI, LK, and LK + RI. (**B**) PCA of different indicators, which were measured in this study, in KN-9204. (**C**) Pearson correlation analyses of different indicators, which were measured in this study, in KN-9204. CK, normal potassium; CK + RI, CK added with *Rhizophagus intraradices* (RI); LK, low potassium; LK + RI, LK added with RI. Gradient color bars denote the correlation coefficient between physiological indexes. The asterisks indicate a significant correlation in the data [*p* < 0.05 (*), *p* < 0.01 (**), *p* < 0.001 (***)].

To understand the correlations between the effects of AMF and changes in various indexes in KN-9204 wheat plants, PCA (principal component analysis) and Pearson correlation analysis were performed in this study. PCA showed that the indices of the four treatments were effectively encapsulated by PC1 (35.9%) and PC2 (32.8%) (Figure 7A). Moreover, different indexes of KN-9204 were also effectively encapsulated by PC1 (86.2%) and PC2 (11.6%). PC1 mostly yielded indexes, such as K content, K accumulation, K utilization efficiency, P and Mg transport coefficients, ASA content, and the K transport-related genes *TaHAK1*, *TaHAK5*, *TaHAK7*, *TaHAK9*, *TaHAK11*, *TaHAK19*, and *TaHAK22*. PC2 yielded K, P, Ca, ASA, and GSH content, K, P, and Mg transport coefficients, fresh weight, the K transport-related genes *TaHAK1*, *TaHAK5*, *TaHAK9*, and *TaHAK19*, and the P transport-related genes of *TaPHT1.6* and *TaPHT1.8* (Figure 7B). The Pearson correlation coefficient showed 113 significantly positive correlations, including for the contents of K and Mg, the K transport coefficient, K accumulation, K utilization efficiency, ASA and GSH content, fresh weight, the *TaHAK1*, *TaHAK5*, *TaHAK7*, *TaHAK11*, *TaHAK19*, and *TaHAK22* in K transport-related genes, the *TaNRT2.2* in N transport-related genes, and the *TaPHT1.3* in the P transport-related gene. It also showed 20 significantly negative correlations among the 29 parameters measured, including for MDA content, the P transport coefficient, the *TaNRT2.4* in the N transport-related gene, and the *TaPHT1.8* and *TaPHT1.6* in P transport-related genes (Figure 7C).

## 3. Discussion

In this study, we determined the effect of AMF-RI on the growth parameters and nutrient accumulation of four wheat varieties under two potassium levels. The results showed that AMF improved the growth of the four wheat varieties, which affected the acquisition and accumulation of nutrients and regulated gene expression. All these findings of this study contribute novel insights into how AMF enhances wheat growth under LK stress treatments, suggesting that applying AMF could be a viable strategy for promoting agricultural sustainability.

### 3.1. AMF Promote Wheat Growth by Regulating the Antioxidant Systems

LK stress disturbs the cellular ion balance and stimulates excessive ROS accumulation, resulting in oxidative damage and slowing plant growth [33,34]. To mitigate LK stress, plants have developed different antioxidant defense systems, which include enzymatic [such as superoxide dismutase (SOD), peroxidase (POX), catalase (CAT), etc.] and non-enzymatic [such as ASA, GSH, etc.] components [35,36,37]. AMF have been reported to alleviate stress damage in various plants. For example, Zhang et al. (2023) found that inoculation with AMF activates the antioxidant enzyme system and improves osmoregulation ability in rice under salt stress [38]. Chen et al. (2024) found that AMF increase the activities of antioxidant enzymes and reduce the content of MDA in Alhagi sparsifolia seedlings under salt stress [39]. Huang et al. (2023) found that inoculation of AMF significantly improves wheat growth attributes under salt stress [20]. In this study, we found that AMF inoculation improved wheat growth and enhanced GSH and ASA contents in wheat plants while decreasing MDA content, especially under LK stress (Figure 2 and Figure 3), suggesting that AMF improve wheat growth by alleviating the oxidative damage of ASA, GSH, and MDA. Moreover, interaction analyses found that GSH contents were significantly different depending on the wheat variety, particularly in KN-9204.

### 3.2. AMF Improve Wheat Growth by Metabolizing K and Other Mineral Elements

AMF can affect nutrient uptake in different plants. It is well known that AMF improve the absorption of phosphorus (P) nutrition in plants by allowing them access to unavailable soil P sources [40,41,42]; AMF also play a role in the uptake of nitrate and ammonium, which are assimilated and transported within the mycelium as arginine [43,44], and this also translate substantially into plants N nutrition. Moreover, AMF positively affect sulfur homeostasis in wheat under drought conditions [45]. However, compared with P and N, few studies have highlighted the roles of AMF in K uptake and transport in different plant species. For example, Xu et al. [46] found that inoculation of AMF increased K concentration in maize to improve plant dry weight. Han et al. [47] found that inoculation of AMF increased shoot and total potassium contents in *Lycium barbarum*, while Yuan et al. [27] found that AMF promoted root K accumulation in sweet potato. To our knowledge, few studies have focused on the effect of AMF on potassium uptake in wheat. Thus, we measured K and several other nutrient elements under normal and LK stress treatments (Figure 4; Table A2). Our results found that the inoculation of AMF increased the nutrient element contents of K, P, Ca, and Mg under CK + RI and LK + RI treatments, especially under LK + RI (Figure 4; Table A2). Interaction analysis in the various wheat varieties found that K content, the K transport coefficient, K accumulation, K utilization efficiency, and P content in the shoots were significantly different at *p* < 0.001; additionally, Ca content in the roots and the Mg translocation coefficient were significantly different at *p* < 0.01. It was noted that Ca and Mg contents in the roots were significantly increased in AK-58, but they were not significantly different in other wheat varieties. All these results indicate that AMF enhance the absorption of K, P, Ca, and Mg nutrient elements, improving wheat growth, and that this is affected by the genotypes of the specific wheat varieties.

### 3.3. AMF Affect Wheat Growth by Affecting the Expression of Genes Related to Mineral Element Absorption and Transport

N, P, and K are the three essential nutrients that are required for plant growth. It has been reported that the abundances of K, P, and N are correlated in spores [47,48]. Similarly, we found that K content, the K transport coefficient, K accumulation, and K utilization efficiency are more positively correlated with P content in KN-9204 than in other wheat varieties. AMF can induce the expression of transporter genes under P-deficient conditions [49,50,51,52,53,54]. Thus, we measured the expression levels of N, P, and K transporter genes in KN-9204 roots under different treatments (Figure 6). The results indicated that the expression levels of the N transporter gene *TaNRT2.2*, the P transporter genes *TaPHT1.3* and *TaPHT1.9*, and the K transporter genes *TaHAK1*, *TaHAK5*, *TaHAK7*, *TaHAK9*, *TaHAK11*, *TaHAK19*, and *TaHAK22* were significantly increased under AMF treatment (Figure 6). Moreover, these genes’ expression levels were more induced in LK + RI than in CK + RI; this was especially the case for K transporter genes (Figure 6). All these results suggest that AMF could regulate K and P transporter genes’ expression to affect the uptake of K, P, and other nutrient elements, thus improving wheat growth.

## 4. Materials and Methods

### 4.1. Experimental Material and Experimental Design

Wheat varieties of Kenong 9204 (KN-9204), Lunxuan 987 (LX-987), Xinmai 26 (XM-26), and Aikang 58 (AK-58) were randomly selected and used in this study. The seeds of wheat were surface-sterilized with 75% ethyl alcohol and washed with ddH_2_O; then, they were germinated for 2 days at 28 °C. Then, these germinated seeds were cultivated in 1/10 Hoagland nutrient solution at 25 °C as previously described [55].

Ten-day-old wheat seedlings, grown in the incubator at 25 °C/16 °C, 16 h/8 h, 60%/75% (day/night) with 12,000 lx, were selected and divided into four groups: (1) control (CK), normal potassium (K) in moist soil, which contained 0.09 g/kg total nitrogen (N), 0.06 g/kg total phosphorus (P), 0.88 g/kg total K, 52.67 mg/kg alkali-hydrolyzable N, 17.57 mg/kg available P, 130 mg/kg available K, 0.63 g/kg soil organic matter, 82% moisture content, and pH 8.46; (2) low potassium (LK) soil, which contained normal N, P, and other physical and chemical characteristics, with low K, 0.85 g/kg total K, and 80 mg/kg available K; (3) CK with arbuscular mycorrhizal fungi (AMF) of *Rhizophagus intraradices* (RI) (CK + RI), 10 g of AMF-RI applied 2 cm from the soil surface in each pot; (4) LK + RI, LK added with RI. To eliminate background contamination of AMF and reduce miscellaneous bacteria, the soil in different treatments was sterilized by high-pressure steam (121 °C, 2 h) [56]. The AMF-RI was supplied from the Institute of Root Biology at Yangtze University, its inoculum contained AMF-infected root segments, spores, and hyphae and was approximately 25–30 AMF/g propagules 1.0 g, and the inoculum was stored at 4 °C under dark conditions. To ensure the purity and activity of RI inoculum, these inoculums were propagated using a maize host plant every three months, as previously described [57]. And they were irrigated twice a week, CK and CK + RI were added with a normal nutrient solution with 6 mmol/L K^+^, which contained macronutrients (1.0 mM Ca(NO_3_)_2_⋅4H_2_O, 0.25 mM NaH_2_PO_4_, 1.0 mM NH_4_NO_3_, 6.0 mM KCl, and 1.0 mM MgSO_4_⋅7H_2_O) and micronutrients (such as EDTA-Fe, H_3_BO_3_, MnSO_4_, ZnSO_4_, CuSO_4_, and (NH_4_)_6_Mo_7_O_24_ [5]); LK and LK + RI were added with potassium deficiency (DK) nutrient solution, which contained 0 mmol/L K^+^. The growth phenotype was recorded every 10 days after treatment. After 30 days, the shoots and roots of wheat plants were harvested to measure the different growth parameters.

### 4.2. Determination of Mycorrhizal Infection Rate

The roots of wheat seedlings were collected and washed, and then they were cut into 1 cm root sections and stained with acid-ink staining, as previously described [58]. Briefly, 100 root segments, from both the main and lateral roots, were randomly selected and used for staining to observe whether they contained AMF structures using the stereomicroscope (Carl Zeiss, Jena, Germany). The mycorrhizal infection rate [(number of AMF roots − number of no AMF roots)/total number of observed roots × 100%] and mycorrhizal dependency index [(total biomass after inoculation of AMF − total biomass without inoculation of AMF)/total biomass without inoculation of AMF × 100%] of wheat roots were calculated, as previously described [38,59].

### 4.3. Determination of the Contents of ASA, GSH, and MDA and the H_2_O_2_ Staining

Fresh wheat leaves (0.2 g) were ground and then added to 0.5% trichloroacetic acid (TCA, purity ≥ 99.0%), as previously described [60]. The contents of malondialdehyde (MDA), ascorbic acid (ASA), and glutathione (GSH) were measured, as previously described [60]. The hydrogen peroxide (H_2_O_2_) staining of wheat leaves using 3,3-diaminobenzidine (DAB) was completed as previously described [61]. After staining, the stained leaves were observed using a stereoscopic microscope (Nikon, Minato City, Japan).

### 4.4. Determination of Mineral Elements

The mineral contents, such as K, P, Na, Mg, and Ca, were measured by inductively coupled plasma mass spectrometry (ICP-MS), (Perkin-Elmer Sciex, Waltham, MA, USA). The element transport coefficient, from root to shoot, and the accumulation and utilization efficiency of K were calculated according to the K content as previously described [62,63,64].

### 4.5. RNA Extraction and Quantitative Real-Time Polymerase Chain Reaction (qPCR)

Total RNA was extracted from roots (0.1 g) using the TransZol RNA Isolation Reagent (Tiangen, Beijing, China), and it was reverse transcribed into cDNA by using the ReverTra Ace qPCR RT Kit (Toyobo, Osaka, Japan). Then, the cDNA was quantified for qPCR analysis by using a QuantStudio 3 (Thermo, Waltham, MA, USA) system as previously described [65]. Briefly, PCR amplification programs were performed as follows: 95 °C for 30 s with one cycle, 95 °C for 10 s, 56 °C for 15 s, and 72 °C for 20 s with 40 cycles; then, the PCR products were analyzed by using a melt curve. The gene relative expression levels were calculated according to the 2^−ΔΔCT^ method as previously described [65]. The wheat glyceraldehyde 3-phosphate dehydrogenase (*GAPDH*) gene was used as an endogenous control gene in this study [66]. All used primers are listed in Table A1. All the primers were amplificated in different concentrations, and their amplification efficiencies were maintained at 92.4–108.0%.

### 4.6. Statistical Analysis

To ensure data normality and homogeneity, all the related data in this study were tested by the Kolmogorov–Smirnov test and Levene’s test. The significant differences among four treatments in one wheat variety or in four wheat varieties were analyzed using a one-way ANOVA test, and the significant differences between two samples, which were from different treatments or varieties, were analyzed by using an LSD post hoc test. The multiple testing corrections were performed by using Tukey’s test and the LSD test. Moreover, the interactions of the variety (V), AMF inoculation (M), and K level (K) were employed by using the Kruskal–Wallis test [67]. All these significant differences were performed by using SPSS 25.0 software. All the data in this study had at least three biological replicates.

## 5. Conclusions

The aim of this study was to evaluate the effectiveness of AMF in promoting wheat growth under low potassium stress treatments. Our results indicate that AMF inoculation improves wheat growth and GSH and ASA contents, while reducing H_2_O_2_ and MDA contents, under low potassium stress treatments. We also found that the inoculation of AMF enhances the uptake of K, P, Ca, and Mg nutrient elements and their uptake as well as the expression of their transporter-related genes. These results demonstrate the potential of AMF to improve crop growth in low-potassium areas by changing oxidative stress indicators, enhancing nutrient element uptake, and regulating transporter-related gene expression. These insights not only enrich our knowledge of AMF’s functions under LK stress treatments but also provide a sustainable agricultural alternative for growing wheat in LK soil.

## Figures and Tables

**Figure A1:**
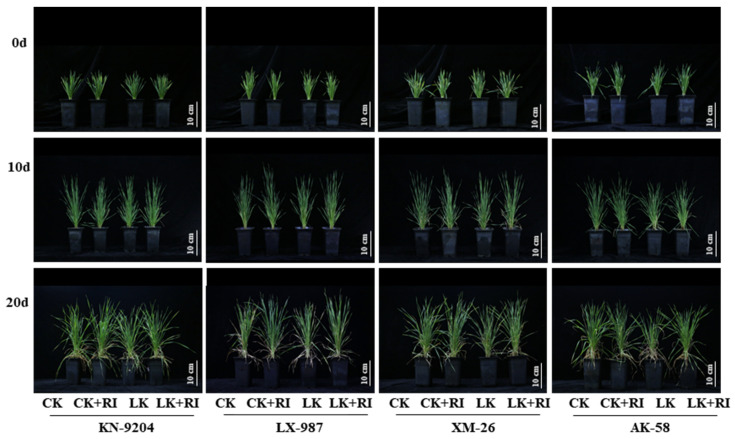
Phenotype effects of AMF-RI colonization on the growth of four wheat varieties under CK and LK treatments at 0 d, 10 d, and 20 d; bar = 10 cm. CK, normal potassium; CK + RI, CK added with *Rhizophagus intraradices* (RI); LK, low potassium; LK + RI, LK added with RI. Four wheat varieties were Kenong 9204 (KN-9204), Lunxuan 987 (LX-987), Xinmai 26 (XM-26), and Aikang 58 (AK-58).

## Data Availability

Data and material of this study are available from the corresponding author upon reasonable request.

## Appendix A

### Appendix A.1

**Table A1:** All primer sequences used in this study.

Gene Name	Primer Sequence (5′-3′)
qPCR-*TaNRT2.2*-F	GCTGCCTGTCGCTCTTGT
qPCR-*TaNRT2.2*-R	CCTTGTTCTTCTCCTCCTCC
qPCR-*TaNRT2.4*-F	TGGACTCGGAGAACAAGG
qPCR-*TaNRT2.4*-R	AGACAAAGCAGGTGAAGAAGG
qPCR-*TaNRT2.5*-F	CTGAAGAGCGCCCATACTACGT
qPCR-*TaNRT2.5*-R	CAGCATAAAGCACCCTCCAAA
qPCR-*TaPHT1.3*-F	CTGCTCACCTACTACTGGCGG
qPCR-*TaPHT1.3*-R	CCCGAAGTCGTTGGCTCC
qPCR-*TaPHT1.6*-F	TTTTTTATGGTCGGAGAGCGTT
qPCR-*TaPHT1.6*-R	CAGCCCTAATTAACCTGGACAACT
qPCR-*TaPHT1.8*-F	GATCTTCAGGGACATCAAGTGGATC
qPCR-*TaPHT1.8*-R	TGAACCCGAGGAACTGGATGG
qPCR-*TaPHT1.9*-F	GAGACCGGCTACTCACGGG
qPCR-*TaPHT1.9*-R	CTAAGCTTCGATGCCATCGTC
qPCR-*TaHAK1*-F	GTTCGAGTCATCCACACCTC
qPCR-*TaHAK1*-R	CACCACACAGATCCCGTAAG
qPCR-*TaHAK5*-F	ACAGGAACCGAAGCAATGTTTG
qPCR-*TaHAK5*-R	GATATGCAGCCTGTCCCATGTA
qPCR-*TaHAK7*-F	CCGGGCTGATCATGAGAGAC
qPCR-*TaHAK7*-R	GTTGATCTGCTCCTCCTCGG
qPCR-*TaHAK9*-F	TCTACAAGAGCACCTTCGCC
qPCR-*TaHAK9*-R	GAGACGTACTTGAGGAGCGG
qPCR-*TaHAK11*-F	CGAGCCTTGGTCTAGTCAGG
qPCR-*TaHAK11*-R	ACCGGGACTGTGTAGACTGG
qPCR-*TaHAK19*-F	AAGACCAGGATGATCCCCAA
qPCR-*TaHAK19*-R	TGCCGAGGATGGTAATTGTG
qPCR-*TaHAK22*-F	GTGAAGCGCTACAAGTACGA
qPCR-*TaHAK22*-R	GATCTTCTCGATGAGGTGCG

### Appendix A.2

**Table A2:** Effects of AMF-RI colonization on the element uptake of four wheat varieties under CK and LK treatments.

Varieties	Treatments	P Content in Shoots (mg/g)	P Content in Roots (mg/g)	P Translocation Coefficient	Mg Content in Shoots (mg/g)	Mg Content in Roots (mg/g)	Mg Translocation Coefficient	Ca Content in Shoots (mg/g)	Ca Content in Roots (mg/g)	Ca Translocation Coefficient
KN-9204	CK	0.10 ± 0.00 c	0.08 ± 0.01 a	1.22 ± 0.01 d	0.14 ± 0.01 a	0.10 ± 0.00 a	1.34 ± 0.02 b	0.32 ± 0.02 a	0.43 ± 0.01 b	0.75 ± 0.01 b
	CK + RI	0.13 ± 0.00 a	0.07 ± 0.00 a	1.93 ± 0.02 b	0.13 ± 0.00 b	0.11 ± 0.01 a	1.11 ± 0.01 d	0.25 ± 0.01 b	0.42 ± 0.02 b	0.59 ± 0.02 d
	LK	0.09 ± 0.00 c	0.05 ± 0.01 b	1.73 ± 0.02 c	0.14 ± 0.01 a	0.11 ± 0.02 a	1.31 ± 0.00 c	0.28 ± 0.03 b	0.32 ± 0.01 c	0.88 ± 0.00 a
	LK + RI	0.11 ± 0.01 b	0.06 ± 0.01 b	1.94 ± 0.01 a	0.15 ± 0.01 a	0.10 ± 0.03 a	1.52 ± 0.02 a	0.31 ± 0.01 a	0.49 ± 0.02 a	0.65 ± 0.01 c
LX-987	CK	0.13 ± 0.01 c	0.06 ± 0.00 b	2.18 ± 0.00 b	0.12 ± 0.01 b	0.09 ± 0.02 b	1.40 ± 0.01 c	0.29 ± 0.02 c	0.36 ± 0.01 b	0.82 ± 0.02 c
	CK + RI	0.20 ± 0.00 a	0.07 ± 0.01 a	2.92 ± 0.03 a	0.11 ± 0.00 b	0.12 ± 0.01 a	0.95 ± 0.03 d	0.17 ± 0.03 d	0.63 ± 0.00 a	0.27 ± 0.03 d
	LK	0.09 ± 0.02 d	0.07 ± 0.00 a	1.35 ± 0.00 d	0.17 ± 0.01 a	0.08 ± 0.02 b	2.10 ± 0.02 a	0.35 ± 0.00 b	0.29 ± 0.01 d	1.22 ± 0.02 a
	LK + RI	0.15 ± 0.01 b	0.08 ± 0.01 a	1.96 ± 0.03 c	0.18 ± 0.02 a	0.11 ± 0.03 a	1.65 ± 0.01 b	0.39 ± 0.01 a	0.33 ± 0.02 c	1.18 ± 0.02 b
XM-26	CK	0.12 ± 0.00 a	0.08 ± 0.01 a	1.65 ± 0.02 d	0.12 ± 0.01 c	0.13 ± 0.02 a	0.95 ± 0.00 c	0.31 ± 0.02 b	0.59 ± 0.01 c	0.52 ± 0.01 b
	CK + RI	0.11 ± 0.01 a	0.05 ± 0.01 b	2.07 ± 0.01 b	0.14 ± 0.02 b	0.14 ± 0.01 a	0.99 ± 0.01 d	0.34 ± 0.01 a	0.70 ± 0.03 b	0.48 ± 0.01 c
	LK	0.10 ± 0.00 b	0.05 ± 0.00 b	1.87 ± 0.01 c	0.15 ± 0.01 b	0.09 ± 0.01 b	1.74 ± 0.02 a	0.37 ± 0.2 a	0.46 ± 0.02 d	0.79 ± 0.02 a
	LK + RI	0.12 ± 0.01 a	0.06 ± 0.01 b	2.10 ± 0.00 a	0.17 ± 0.01 a	0.13 ± 0.02 a	1.30 ± 0.03 b	0.37 ± 0.03 a	0.84 ± 0.01 a	0.45 ± 0.03 c
AK-58	CK	0.10 ± 0.00 c	0.05 ± 0.01 b	1.84 ± 0.02 b	0.11 ± 0.01 a	0.09 ± 0.03 b	1.16 ± 0.01 b	0.23 ± 0.01 b	0.23 ± 0.01 c	1.01 ± 0.01 b
	CK + RI	0.12 ± 0.01 b	0.07 ± 0.00 a	1.58 ± 0.03 d	0.12 ± 0.00 a	0.13 ± 0.01 a	0.94 ± 0.02 d	0.28 ± 0.02 a	0.64 ± 0.02 b	0.43 ± 0.01 c
	LK	0.12 ± 0.00 b	0.07 ± 0.01 a	1.78 ± 0.01 c	0.13 ± 0.02 a	0.09 ± 0.02 b	1.50 ± 0.01 a	0.28 ± 0.03 a	0.25 ± 0.03 c	1.13 ± 0.01 a
	LK + RI	0.18 ± 0.02 a	0.06 ± 0.01 a	2.90 ± 0.00 a	0.13 ± 0.01 a	0.13 ± 0.01 a	0.98 ± 0.00 c	0.18 ± 0.01 c	0.72 ± 0.03 a	0.26 ± 0.01 d
KN-9204		A	A	A	A	A	A	A	A	A
LX-987		B	A	A	A	A	A	A	A	A
XM-26		AB	A	A	A	A	B	A	B	A
AK-58		AB	A	A	A	A	A	A	A	A

K, normal potassium; CK + RI, CK added with *Rhizophagus intraradices* (RI); LK, low potassium; LK + RI, LK added with RI. Four wheat varieties were Kenong 9204 (KN-9204), Lunxuan 987 (LX-987), Xinmai 26 (XM-26), and Aikang 58 (AK-58). Data present means ± SE (*n* = 3). Data present means ± SE (*n* = 3). Different letters are used to indicate significant differences; the small letters indicate differences between treatments within each variety, and the capital letters indicate differences between varieties (Tukey’s test, *n* = 3, *p* < 0.05).

**Table A3:** Significant differences in variety, AMF inoculation and K level.

Measurement Index	V	M	K	V × M	V × K	M × K	V × M × K
P content in shoots	***	***	***	***	***	***	***
P content in Roots	ns	ns	***	***	***	***	***
P transport coefficient	ns	***	ns	***	***	***	***
Mg content in shoots	ns	ns	ns	ns	ns	ns	ns
Mg content in roots	ns	***	*	***	**	**	**
Mg translocation coefficient	**	***	***	***	***	ns	ns
Ca content in shoots	ns	ns	***	*	***	*	***
Ca content in roots	**	***	***	***	***	*	***
Ca translocation coefficient	ns	***	***	***	***	ns	***

Asterisks indicate significant differences in variety (V), AMF inoculation (M), K level (K), and their interactions [Kruskal–Wallis test, *n* = 4, *p* < 0.05 (*), *p* < 0.01 (**), *p* < 0.001 (***) and *p* > 0.05 (ns)].
